# Cloning and Functional Characterization of Cold-Inducible MYB-like 17 Transcription Factor in Rapeseed (*Brassica napus* L.)

**DOI:** 10.3390/ijms24119514

**Published:** 2023-05-30

**Authors:** Dan Luo, Ali Raza, Yong Cheng, Xiling Zou, Yan Lv

**Affiliations:** Key Laboratory of Biology and Genetic Improvement of Oil Crops, Oil Crops Research Institute, Chinese Academy of Agricultural Sciences (CAAS), Ministry of Agriculture, Wuhan 430062, China

**Keywords:** abiotic stress, canola, gene overexpression, low temperature, osmoprotectants, transcriptome analysis

## Abstract

Rapeseed (*Brassica napus* L.) is an important crop for edible oil, vegetables, and biofuel. Rapeseed growth and development require a minimum temperature of ~1–3 °C. Notably, frost damage occurs during overwintering, posing a serious threat to the productivity and yield of rapeseed. MYB proteins are important transcription factors (TFs) in plants, and have been proven to be involved in the regulation of stress responses. However, the roles of the MYB TFs in rapeseed under cold stress conditions are yet to be fully elucidated. To better understand the molecular mechanisms of one MYB-like 17 gene, *BnaMYBL17*, in response to low temperature, the present study found that the transcript level of *BnaMYBL17* is induced by cold stress. To characterize the gene’s function, the 591 bp coding sequence (CDS) from rapeseed was isolated and stably transformed into rapeseed. The further functional analysis revealed significant sensitivity in *BnaMYBL17* overexpression lines (*BnaMYBL17*-OE) after freezing stress, suggesting its involvement in freezing response. A total of 14,298 differentially expressed genes relative to freezing response were found based on transcriptomic analysis of *BnaMYBL17*-OE. Overall, 1321 candidate target genes were identified based on differential expression, including Phospholipases C1 (*PLC1*), FCS-like zinc finger 8 (*FLZ8*), and Kinase on the inside (*KOIN*). The qPCR results confirmed that the expression levels of certain genes showed fold changes ranging from two to six when compared between *BnaMYBL17*-OE and WT lines after exposure to freezing stress. Furthermore, verification indicated that *BnaMYBL17* affects the promoter of *BnaPLC1*, *BnaFLZ8,* and *BnaKOIN* genes. In summary, the results suggest that *BnaMYBL17* acts as a transcriptional repressor in regulating certain genes related to growth and development during freezing stress. These findings provide valuable genetic and theoretical targets for molecular breeding to enhance freezing tolerance in rapeseed.

## 1. Introduction

Rapeseed (*Brassica napus* L.) is the largest oil crop in China, accounting for about 20% of world production [1]. With the development of society, rapeseed is not only intended to meet oil and vegetable, flower (leisure and sightseeing), honey, feeding, and fertilizer needs, but also has certain nutritional and health value. Climate change events, such as cold stress, greatly threaten rapeseed production globally [2,3,4]. Around the Yangtze River basin of China, late harvest in double-cropping rice areas has resulted in delayed sowing dates for rapeseed, leading to decreased germination rate, seedling rate, or survival rate during the vegetative growth period, and decreased pollen fertility, segmented seed setting, yield, and quality during the reproductive growth period [1,2,5]. Therefore, improving cold tolerance and developing superior-quality and high-yielding cultivars depends on the thorough investigation and elucidation of the mechanisms involved in cold tolerance in rapeseed.

In plants, cold stress is mainly divided into chilling (0–15 °C) and freezing (<0 °C) [6,7,8]. Chilling stress affects the growth and development of plants by repressing plasma membrane fluidity and enzyme activity, meanwhile inducing the accumulation of reactive oxygen species (ROS) [6,7,9]. Freezing temperatures lead to cellular dehydration due to extracellular ice formation, resulting in mechanical damage as well as possible plant death [8,10,11]. Cold stress also destroys cell membrane and ion balance and blocks the photosynthetic or metabolic processes at the cellular level [12]. To date, plants have evolved a set of physiological, biochemical, and molecular responses to cope with cold stress [6,7,13]. For instance, plants increase activities of antioxidant enzymes, such as catalase (CAT), superoxide dismutase (SOD), ascorbate peroxidase (APX), and peroxidase (POD), by regulating the expression of antioxidant-related genes to remove intracellular ROS; thus, reducing oxidative damage [6,9,14,15]. Plants also exhibit an increased synthesis of polyphenols such as phenolic acids and flavonoids under abiotic stress conditions, which also help the plants to cope with environmental constraints, including chilling stress [16].

Transcription factors (TFs) act as trans-acting elements through their specific binding to *cis*-acting elements in the promoters of target genes and play central roles in regulating the expression of downstream genes [17,18]. The MYB TFs were first discovered in the avian leukosis virus, v-MYB virus [19], which are named for R1R2R3-MYB (3R-MYB), R2R3-MYB (2R-MYB), 4R-MYB, and MYB-related based on an MYB domain containing one to four imperfect repeats of 52 amino acids [20]. In the *Arabidopsis* MYB family, there were 198 members [21], while in rapeseed, there were 251 MYB-related proteins that were classified into five distinct clades [22]. The widespread members of the MYB family have diverse roles in multiple pathways. For instance, previous studies indicated that *MYB* genes are involved in plant primary and secondary metabolism, such as flavonoid, anthocyanin, glucosinolate biosynthesis [23,24], growth and development [25,26,27], and responses to diverse abiotic stresses [18,20,25]. MYB TFs play critical roles in cold tolerance by regulating C-repeat binding factor (*CBF*) genes. Among these, *AtMYB15* repressed the *CBF* genes by direct binding and regulated freezing tolerance in *Arabidopsis* [28], and *AtMYB96* activates drought tolerance through indirect upregulation of *CBF* expression [29], while *OsMYB30* works independently of *CBF* genes, and it negatively regulates the cold tolerance in rice by repressing the starch breakdown and the content of maltose [30]. *BnaMYB78* modulates ROS-dependent cell death in tobacco (*Nicotiana benthamiana*) by regulating the transcription of ROS- and defense-related genes [21]. Another rapeseed *MYB* gene (*BnaMYB111L*) activates the expression of genes, involved in ROS generation, cell death, and defense responses [31]. Moreover, overexpression of the MYB-related gene *BnMRD107* improves the osmotic tolerance in rapeseed seedlings [22]. Recent genome-wide association and transcriptome analysis in rapeseed have shown that MYB TFs regulate salt stress tolerance [32]. A recent transcriptome study showed that several *MYB* genes were differentially expressed when rapeseed was exposed to freezing stress [33]. Previous studies showed that post-translational adjustments play a critical role in controlling the activity and strength of the MYB15 protein. MPK6 interacts with MYB15 and phosphorylates it at Ser-168, leading to the reduced kinship of MYB15 protein for binding to the *CBF3* promoter, whereas the overexpression of *MYB15*, which cannot be phosphorylated by *MPK6,* leads to reduced *CBF* expression levels and increased sensitivity to freezing stress, as compared to plants overexpressing *MYB15* [34]. However, there is almost no report on the molecular mechanism of *MYB* genes in regulating cold stress tolerance in rapeseed.

Our previous study identified an MYB-like gene in rapeseed, *BnaMYBL17*, which is responsive to cold stress [35], and provided valuable information to better understand and evaluate the function of *MYB* genes in rapeseed. Therefore, the expression patterns of *BnaMYBL17* in response to various stresses and different tissues were analyzed. The *BnaMYBL17* gene was overexpressed in rapeseed to fully uncover its potential in regulating freezing tolerance. Moreover, the transcriptome analysis was conducted to screen the downstream differentially expressed genes (DEGs), and a yeast one-hybrid (Y1H) assay was performed to confirm the interaction between BnaMYBL17 and promoters of candidate target genes. In short, the present study reports that *BnaMYBL17* acts as a negative regulator of freezing tolerance by modulating the expression of target genes and affecting their promoter regions.

## 2. Results

### 2.1. Characterization of BnaMYBL17

**Figure 1:**
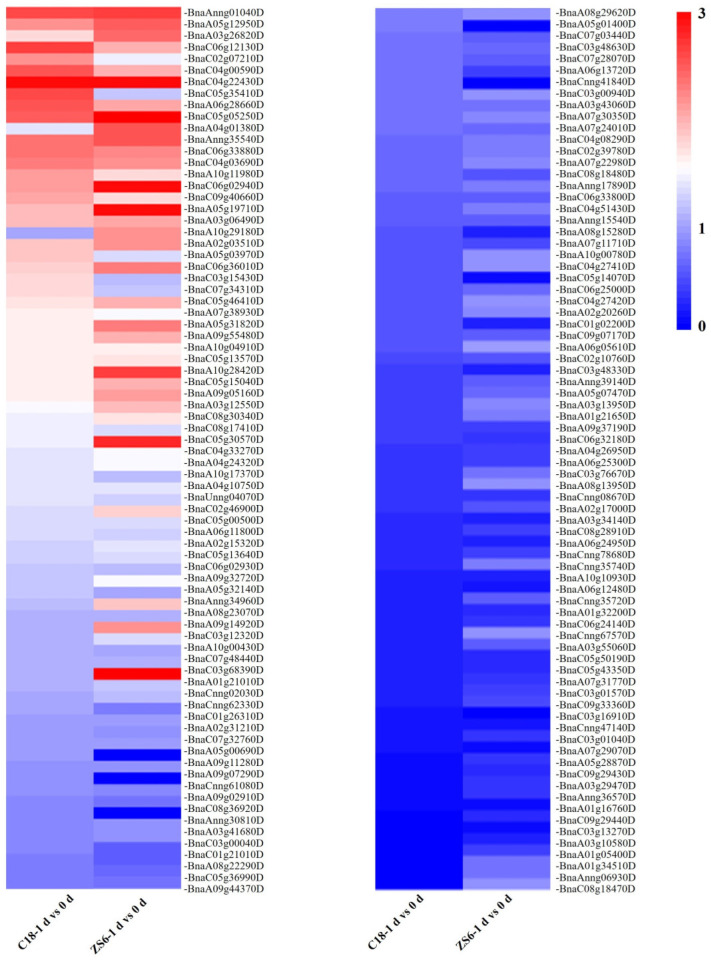
Transcriptome analysis of *BnaMYBL* family genes. Notes: ZS6 is a cold-sensitive rapeseed type ‘Zhongshuang 6’; C18 is a cold-tolerant rapeseed type ‘1801’.

**Figure 2:**
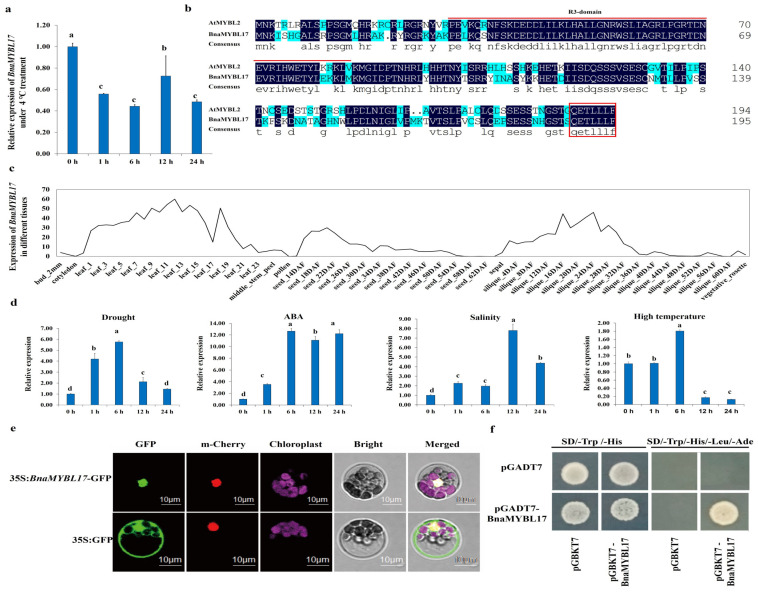
(**a**) Detection of the expression of the gene *BnaMYBL17* under −4 °C treatment. (**b**) Amino acid alignment of the gene *BnaMYBL17* with its homologue gene AtMYBL2 in *Arabidopsis*. Red lines/box represents to R3 domain. (**c**) Expression of *BnaMYBL17* in different tissues. (**d**) Expression analysis of *BnaMYBL17* under drought, ABA, salinity, and high-temperature treatment. Bars indicate the SD of three biological replicates. Statistical analysis is determined by the least significant difference (LSD) test (*p* < 0.05). (**e**) Subcellular localization of *BnaMYBL17* in *Arabidopsis* protoplasts. The pUC19-35S-GFP plasmid was a control. The *Arabidopsis*-AtHY5-carried m-Cherry label was a nuclear location marker. The GFP and mCherry signals were observed by a confocal microscope. Excitation/emission wavelengths of GFP and mCherry were 488 nm to 540 nm and 552 nm to 640 nm, respectively. Here, the blue color indicates the chlorophyll signal. Bars = 10 μm. (**f**) Transactivation experiment with BnaMYBL17 in yeast. Growth of yeast cells (strain AH109) transformed with pGBKT7-BnaMYBL17 and pGADT7 vector on the SD/-Trp/-His medium and SD/-Ade/-His/-Leu/-Trp medium; the transformation with pGBKT7 and pGADT7 vector was used as the positive control. In Figure 2a,d, different alphabets/letters on bars represent statistically significant at *p* < 0.05.

Our previous results showed significant differences in physiological and phenotypic characteristics in a cold-sensitive variety Zhongshuang 6 (ZS6) compared to a tolerant variety 1801 (C18) [35,36,37]. Therefore, we screened out 36 significantly downregulated and 14 upregulated DEGs belonging to the MYB family, having > log_2_ (FC) values (Figure 1). The evolutionary trees of this subfamily have been reported in previous studies [22]. The qRT-PCR results confirmed that *BnaMYBL17* was downregulated in response to cold stress, which was consistent with the RNA-seq results (Figure 2a). Sequence alignment analysis showed that *BnaMYBL17* has the typical structural characteristics of an MYB-related protein family containing only one R3-domain of 55 amino acids, which showed 62.6% similarity to the amino acid sequence of AtMYBL2. In addition, its terminal contains a six-amino acid (TLLLFR) motif that appears to be a novel repression motif [38] (Figure 2b).

In order to explore the expression of *BnaMYBL17* gene in rapeseed, its transcript patterns during different developmental stages (cotyledon, root, stem, leaf, shoot apical meristem (SAM), overleaf, flower, ovary, and seed at 14, 18, 22, 26, 30, 34, 38, 42, 46, 50, 54, 58, and 62 DAP, and silique wall at 4, 8, 12, 16, 20, 24, 28, 32, 36, 40, 44, 48, 52, 56, and 60 DAP) were examined. The transcriptome data showed that *BnaMYBL17* is expressed in all tissues, especially in stems and leaves (Figure 2c). To clarify the potential roles of *BnaMYBL17* in responses to various abiotic stresses, its expression pattern was investigated under drought, salt, heat stress, and ABA treatments (Figure 2d). Under high-temperature and drought conditions, the expression of *BnaMYBL17* reached its maximum at 6 h after treatment and then showed a rapid downward trend. In contrast, the expression of *BnaMYBL17* under ABA and salt stress was significantly higher than before treatment. The above outcomes indicated that multiple stresses may induce the expression level of *BnaMYBL17*, suggesting its vital role in abiotic stress regulation in rapeseed.

To determine the subcellular localization of *BnaMYBL17* protein, its coding sequence (CDS) was fused with the N-terminus of the green fluorescent protein (*GFP*) gene. The results indicated that *BnaMYBL17* was able to co-localize with the reported TF AtHY5 [39] in the cell nucleus (Figure 2e). Furthermore, the Y1H experiments confirmed that the full length of *BnaMYBL17* exhibited self-activation, as the yeast AH109 cells transformed with the pGBKT7-BnaMYBL17 and pGADT7 grew well on the SD/-Trp/-His medium and SD/-Ade/-His/-Leu/-Trp medium (Figure 2f).

### 2.2. Overexpressing BnaMYBL17 Reduced Freezing Tolerance

**Figure 3:**
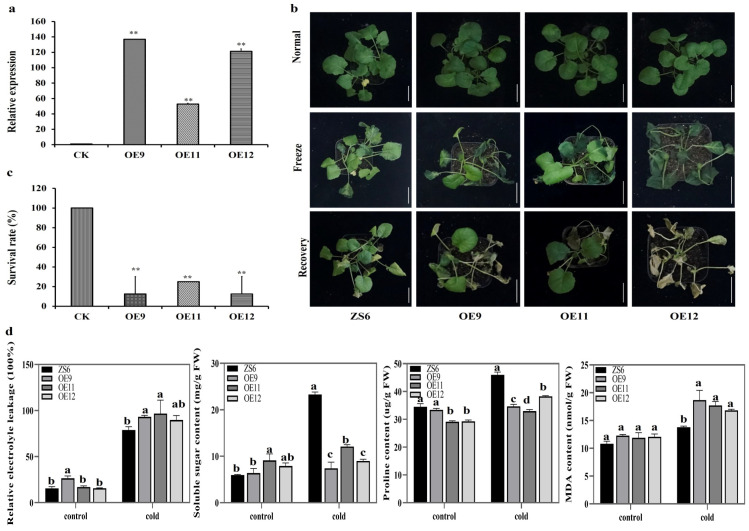
The OE lines were obtained and identified by qRT-PCR, phenotypic, survival rate, physiological, and biochemical indicators assessment in wild type and three *BnaMYBL17* OE lines before and after treatment at −4 °C for 4 h, and recovered for a week WT: wild l type ‘ZS6’; OE9, OE11, and OE12: BnaMYBL17 overexpressing ‘ZS6’ lines. (**a**) Three *BnaMYBL17* OE lines (OE9, OE11, and OE12) were obtained and identified by qRT-PCR. The *ACTIN* gene in rapeseed is used as an internal control. (**b**) Phenotypes of WT plants and transgenic lines before cold treatment, after cold treatment at −4 °C for 4 h, and recovery for one week under normal growth conditions. Bar indicates 5 cm scale. (**c**) Survival statistics. (**d**) Determination of electrolyte leakage, soluble sugar content, malondialdehyde, and proline content. Error bars refer to ± SD (n = 3). Significance was analyzed by the method of least significant difference (LSD) (* *p* < 0.05; ** *p* < 0.01) with SPSS software (version 13.0). In Figure 3d, different alphabets/letters on bars represent statistically significant at *p* < 0.05.

To further investigate the function of *BnaMYBL17*, its CDS was cloned into pCAMBIA1300 vector under the control of 35S promoter, and the resultant vector was then transformed into rapeseed via the agrobacterium-mediated transformation. Three *BnaMYBL17* OE lines (OE9, OE11, and OE12) were obtained and identified by qRT-PCR (Figure 3a). These lines were placed in the incubator and treated at −4 ℃ for 4 h at four-leaf stages, and then were cultured for a week under normal conditions to observe their growth under freezing stress. It was found that the WT plants were slightly wilted after cold treatment, and the leaves of *BnaMYBL17* OE lines were severely wilted (Figure 3b). The growth vigor of seedlings was determined by their survival rate, which was significantly higher in the WT plants than that in OE lines (Figure 3c).

To obtain further insights, several physiological and biochemical indicators were measured before and after cold stress. Compared to the WT plants, the *BnaMYBL17* OE lines presented higher relative electrolyte leakage (REL) in response to freezing stress (Figure 3d), suggesting that the stress-induced plasma membrane damage of overexpression (OE) lines was more serious. On the other hand, there was no significant difference in soluble sugar content between WT and OE lines before treatment (Figure 3d). However, after treatment, the soluble sugar content of OE lines was significantly lower than that of WT plants, which was consistent with the proline (Pro) content investigation (Figure 3d). The cell membrane damage caused by stresses is typically associated with increased malondialdehyde (MDA) content, which is one of the final products of peroxidation of unsaturated fatty acids in phospholipids, making MDA an indicator of plant cold tolerance. In agreement with their freezing tolerance, there was higher MDA content in the OE lines when compared to the WT plants after stress treatment (Figure 3d), indicating that *BnaMYBL17* negatively regulates the freezing tolerance of rapeseed at the seedling stages.

### 2.3. Identification of BnaMYBL17-Regulated COR Genes by Transcriptome Analysis

**Figure 4:**
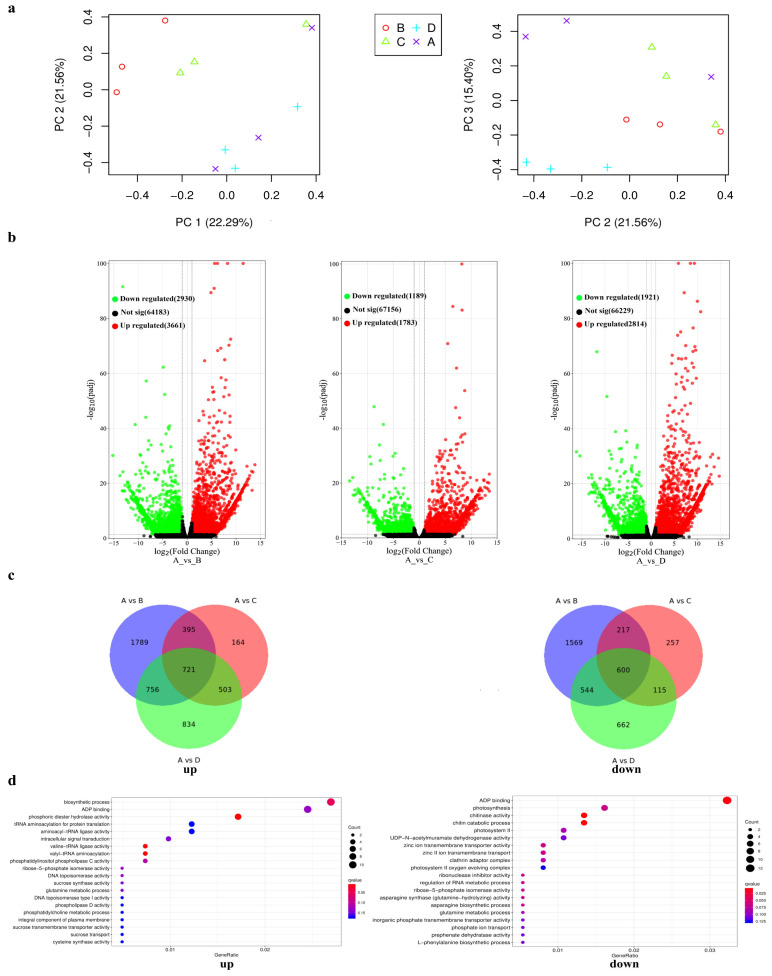
RNA-seq analysis of WT plants and *BnaMYBL17* OE lines exposed to freezing stress. (**a**) Principal component analysis. (**b**) DEGs were detected by comparison of the RNA-seq data of the WT treated vs. the three OE lines, respectively. (**c**) Venn diagram analysis. (**d**) Gene ontology (GO) analysis. In Figure 4a, A stands for control group wild type ‘ZS6’; B stands for *BnaMYBL17* overexpressing ‘ZS6’ lines OE9; C stands for *BnaMYBL17* overexpressing ‘ZS6’ lines OE11; D stands for *BnaMYBL17* overexpressing ‘ZS6’ lines OE12.

To identify the potential targets of *BnaMYBL17* responsible for the freezing response, RNA-seq analysis of WT plants and *BnaMYBL17* OE lines was performed under freezing stress. A principal component analysis (PCA) was first performed, and it verified the repeatability among the replicates (Figure 4a). Moreover, the qRT-PCR-based expression trends of six randomly selected genes showed a strong correlation with the RNA-seq results, indicating the reliability of our RNA-seq datasets (Figure S1). We compared the RNA-seq data of the WT plants with the three OE lines. A total of 14,298 DEGs were detected, including 3661 upregulated and 2930 downregulated genes in OE9, 1783 upregulated and 1189 downregulated genes in OE11, and 2814 upregulated and 1921 downregulated genes in OE12 (Figure 4b). Subsequently, Venn diagram analysis revealed that 721 overlapping upregulated DEGs were shared among the three comparisons, whereas 600 downregulated DEGs were common (Figure 4c).

Moreover, to narrow down the target genes regulated by *BnaMYBL17*, a correlation analysis between the DEGs’ expression levels and *BnaMYBL17*’s overexpression levels was performed using SPASS software (version 13.0). The DEGs with correlation coefficients > 0.9 or < −0.9 were explored as potential targets. Therefore, all upregulated or downregulated DEGs in the three OE lines uncovered a total of 31 and 14 common genes that were upregulated and downregulated by *BnaMYBL17* under freezing conditions (Table S2). To further specify the biological processes, gene ontology (GO) analysis of potential targets was performed (Figure 4d). As shown in Figure 4, the upregulated potential targets were enriched in biological processes involved in the biosynthesis process, ADP binding, phosphoric diester hydrolase activity, tRNA aminoacylation for protein translate, etc., and downregulated potential targets were mainly enriched in ADP binding, photosynthesis, chitinase activity, etc. (Figure 4d).

### 2.4. BnaMYBL17 Improves Freezing Sensitivity by Upregulating the Target Genes

**Figure 5:**
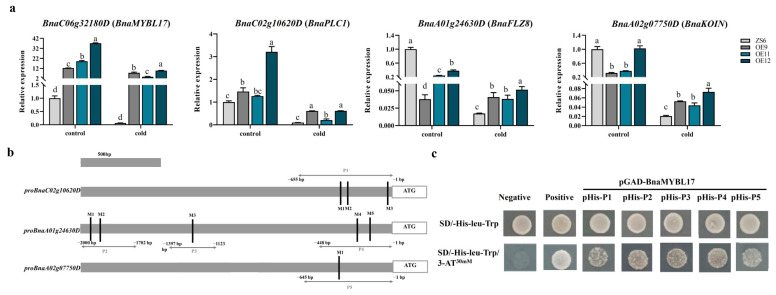
(**a**) Relative expression level analysis of potential targets. Error bars refer to ±SD (n = 3). Significance was analyzed by the method of least significant difference (LSD) (* *p* < 0.05; ** *p* < 0.01) with SPSS software (version 13.0). Different alphabets/letters on bars represent statistically significant at *p* < 0.05. The *ACTIN* gene in rapeseed is used as an internal control. (**b**) Motif analysis of potential targets. (**c**) Yeast one-hybrid assays analysis. Growth of yeast cells (strain Y187) transformed with pGADT7Rec-BnaMYBL17 and the pHIS2.1 vectors with the putative MYB binding sequences on the SD/-Trp/-His medium and SD/-Trp/-Leu/-His selection medium containing 30 mM 3-AT. The transformation with pGADT7Rec-P53 and pHIS2.1-P53 vector was used as the positive control, and the transformation with pGADT7Rec and pHIS2.1 vector was used as the negative control. P1–P5 refer to the five segments containing MYB binding sites in the promoters of the three genes intercepted.

Based on enriched GO terms, *BnaC02g10620D*, *BnaA01g24630D,* and *BnaA02g07750D* were upregulated by *BnaMYBL17*. *BnaC02g10620D* encodes one phospholipase C (*BnaPLC1*), known as a critical enzyme that plays an important role in various signal-transduction pathways in eukaryotic cells [40]. *BnaA01g24630D* encodes FCS-like zinc finger 8 (*BnaFLZ8*), a critical factor in balancing growth and stress-response trade-offs through coordinating the TOR and SnRK1 signaling in plants [41]. *BnaA02g07750D* encodes leucine-rich repeat receptor-like (LRR) proteins and is highly similar to AtKOIN, which is crucial in the transduction of various plant environmental and developmental signals [42]. The upregulation of *BnaPLC1*, *BnaFLZ8,* and *BnaKOIN* was confirmed in the *BnaMYBL17* OE lines compared with the WT after cold treatment (Figure 5a). Motif analysis identified a set of MYB recognition site (CAACGG) in the promoter regions of these three genes, suggesting they are potential targets of *BnaMYBL17* (Figure 5b). To test this possibility, Y1H assays were performed for four promoter fragments (P1–P5), and the current study showed that *BnaMYBL17* interacted with the P1 fragments of *BnaC02g10620D* (*BnaPLC1*), P2–P4 of *BnaA01g24630D* (*BnaFLZ8*), and P5 of *BnaA02g07750D* (*BnaKOIN*) in yeast strain Y187 (Figure 5c), suggesting that *BnaMYBL17* negatively regulates transcription of the two target genes by binding to their promoter.

## 3. Discussion

Rapeseed, as an overwintering crop, is mainly planted in the south of the Yangtze River, which is less sensitive to cold stress [1,2,3]. However, the sowing time for rapeseed is postponed due to the delayed harvest of late rice. Consequently, the rapeseed seed emergency, plant growth, and yield are affected by various abiotic stresses, including cold stress [1,2,3,4]. Therefore, it is vital to elucidate the underlying genetic and molecular basis of cold sensitivity in rapeseed through physiological and molecular methods to fast-track rapeseed production and enlarge its sowing area in the ‘rice–rice–rapeseed’ triple-cropping system.

MYB proteins can be divided into different classes depending on the number of MYB repeats. One class comprises proteins with one or a partial MYB repeat, designated as MYB-related (MYBL or MYBR), that are likely to have evolved from *R2R3-MYB* genes, and are mainly involved in controlling cellular morphogenesis and secondary metabolism control [20]. *MYBL2*, which includes one R3-type MYB repeat and belongs to the MYBL class, is a transcriptional repressor in the biosynthesis of anthocyanin, and inhibits the transcriptional activation of the MBW (MYB–bHLH–WDR) complex through its binding to bHLH subunits which activates the expression of anthocyanin-specific genes [24]. Multiple studies have now shown that MYBL proteins respond to abiotic stresses. An MYB-like gene from Amur grape (*Vitis amurensis*), designated as *VaAQUILO*, is induced by cold stress. Its overexpression significantly improves cold stress tolerance in both transgenic *Arabidopsis* and in Amur grape calli [43]. Two MYBL genes (*MYBS1* and *MYBS2*) of *Arabidopsis* have been shown to have opposite roles in sugar signaling mechanisms [44]. The expression of *OsMYB48-1* was strongly induced by drought, ABA, H_2_O_2_, and dehydration while being slightly induced by high salinity and cold treatment [45]. While its overexpression in rice significantly improves tolerance to drought and salinity stresses, further evidence indicated that *OsMYB48-1* positively functions in drought and salinity tolerance by regulating stress-induced ABA synthesis [45]. The present study also observed similar results, where the expression level of the *BnaMYBL17* gene was strongly induced by ABA, drought, salinity, and high temperature (Figure 2). *OsMYBR1* has been reported to be induced by drought and cold stresses at various developmental stages, and its overexpression enhances drought stress tolerance and decreases sensitivity to ABA in rice [46]. In orchid (*Phalaenopsis equestris* and *Dendrobium officinale*), 15 out of the 42 *MYBR* genes were modulated by cold stress, among which nine were upregulated and three genes were downregulated [47], suggesting the positive role of *MYB* genes in cold stress regulation. Overall, these studies suggest that MYB TFs play a positive role in regulating a diverse set of plant reactions to abiotic stresses, such as drought, cold, salinity, and ABA treatments. It is worth noting that 65 *MYBL* genes, including *BnaMYBL17* were downregulated in the two rapeseed varieties after cold stress (Figure 1). We then determined that *BnaMYBL17* negatively regulates freezing tolerance in rapeseed by modulating the expression of target genes. Hence, it is important to identify the transcriptional regulatory mechanism of *BnaMYBL17* in the freeze-stress response. The current study showed that *BnaMYBL17* interacted with the promoter fragments of *PLC1*, *FLZ8,* and *KOIN* in yeast strain Y187 (Figure 5c), suggesting that *BnaMYBL17* negatively regulates transcription of the three target genes.

Until now, sets of MYB TFs involved in cold tolerance have been reported to be adversely altered [48]. For instance, MYB30 interacts with MYB96, which is involved in ABA signaling; however, these two TFs have opposite functions in ABA-mediated seed germination inhibition, suggesting a finetuned mechanism for the coregulation of MYB TFs [48]. In addition to positive regulators of cold tolerance, such as *AtMYB96* [49], distinct MYB TFs act sequentially and complementarily to adapting cold stress. Therefore, it is logical that plants downregulate negative regulators after cold stimulation, which accordingly protects plants against cold stress [25]. In pepper (*Capsicum annuum* L.), *CaMYB306* acts as a negative regulator of cold tolerance by suppressing the antioxidant defense system and repressing the expression of the cold-related gene *CaCIPK13* [50]. *AtMYB15* loss of function mutants increase cold tolerance by elevating *CBF* gene expression [28]. Similarly, *AtMYB14* is downregulated by cold stress, and the knockdown plants of *AtMYB14* increase the freezing tolerance, in which the *CBF* genes and the downstream genes are also induced to a much higher level [51]. In the current study, cold stress greatly repressed *BnaMYBL17* expression in WT plants, and the expression levels of downstream genes, including *PLC1*, *FLZ8*, and *KOIN*, were remarkably upregulated in OE plants under cold stress. Inconsistent with *AtMYB14*, the transactivation activity assays in yeast revealed that *BnaMYBL17* might be a transcriptional activator. It seems conflicting but is not unusual in plants. *WRKY48* is a transcriptional activator that represses plant basal defense [52]. This evidence indicated that *BnaMYBL17* played a negative role in cold tolerance, such as *CaMYB306*, *AtMYB15*, or *AtMYB14*; nevertheless, the downstream targets of MYB proteins are very different, which requires more investigation in the future.

**Figure 6:**
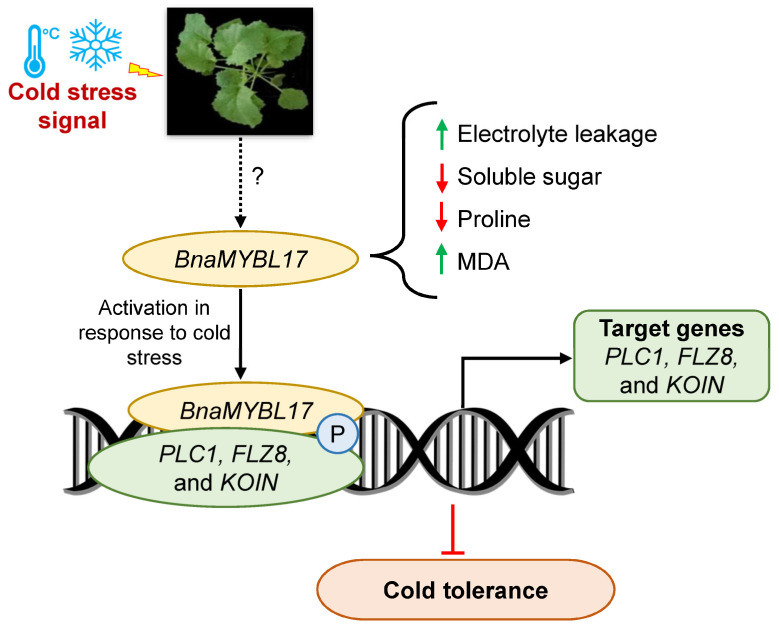
A proposed mechanism model for *BnaMYBL17*-mediated cold stress tolerance in rapeseed. After receiving cold stress signal by the rapeseed plant, *BnaMYBL17* is repressed. The *BnaMYBL17* gene functions as a transcriptional regulator and binds to the promoters of target genes, including *PLC1*, *FLZ8*, and *KOIN*. Thus, it regulates the expression levels of these target genes. Furthermore, the physiological analysis indicates that overexpression transgenic plants showed increased electrolyte leakage and MDA contents while having reduced contents of soluble sugar and proline, suggesting the downstream role of *BnaMYBL17* gene in cold tolerance. These discoveries indicate that *BnaMYBL17* plays a negative regulatory function in the freezing response of rapeseed by controlling the expression of target genes involved in growth and development. In short, the anticipated mechanism delivers insights into the molecular mechanisms underlying cold stress response/tolerance in rapeseed. It also highlights *BnaMYBL17* as a possible target for future molecular breeding approaches to improve freezing tolerance in rapeseed.

Phospholipases C (PLC) hydrolyzes the phosphodiester bond on the glycerol side of phospholipids to produce diacylglycerol (DAG) and a phosphorylated head group. Numerous functional analyses of plant PLCs have strongly supported their role in stress response and plant development [53,54,55]. *Arabidopsis* plants overexpressing *AtPLC4* exhibit hypersensitivity to salt stress, and the corresponding mutants are insensitive; therefore, *AtPLC4* negatively regulates salt tolerance in *Arabidopsis* [56]. Overexpression of *PLC5* decreases stomatal aperture and improved drought tolerance in *Arabidopsis* [57], as well as maize *ZmPLC1*, rapeseed *BnPLC2*, and *Nicotiana tabacum* (tobacco) PLCδ [58,59,60]. However, the exact function of PLCs in cold stress response is unknown. As TOR is an important regulator of seedling development, a previous study investigated the role of *FLZ8* in growth and development, and *flz8* mutants showed enhanced growth with significantly longer primary roots and increased lateral root number and biomass; thus, *FLZ8* represses meristem hyperactivation, leading to limited growth [41]. Until now, more than 400 plant-receptor-like kinases (RLKs) have been detected in *Arabidopsis*. Among them, the LRR-RLKs family is the largest, which includes 216 members.; a set of LRR-RLKs have been reported to function in plant cell signal perception and transmission during plant growth and development [61]. KOIN was introduced as a candidate protein involved in the regulation of ground tissue cell divisions, as KOIN represses root cell proliferation in *Arabidopsis*, and its mutant displayed large roots than WT plants [62]. Here, the present results showed that *BnaKOIN* was upregulated in *BnaMYBL17* OE lines compared with the WT after cold treatment (Figure 5a). It is worth noting that *PLC5* also reduces primary and secondary root growth, and stunted root hairs [63]. However, more studies will be needed to identify the involvement of the three targets in cold stress response. Thus, we propose that *PLC1*, *FLZ8,* and *KOIN* confer freezing sensitivity through limiting growth that converges at *BnaMYBL17*-mediated targeting (Figure 6). Considering *BnaPLC1*, *BnaFLZ8,* and *BnaKOIN* in suppressing growth, these genes may balance growth and cold stress response in rapeseed. In future studies, identifying the precise functions of these target genes will be critical to further untangling the negative mechanisms mediated by *BnaMYBL17*.

## 4. Materials and Methods

### 4.1. Plant Materials, Growth Conditions, and Stress Treatments

The semi-winter rapeseed type, Zhongshuang 6 (ZS6), was used for genetic transformation, gene expression analysis, and physiological measurements under cold stress. The germinated seeds of ZS6 were transplanted into pots (10 cm × 10 cm) in the plant growth room with 16 h light/8 h dark cycle at 23 °C. The pots were filled with a 1:1:1 mixture of peat vermiculite: moss: perlite. The seedlings were then subjected to continuous −4 °C in the camber for 4 h at the four-leaf stage. The leaves were harvested before treatment, 4 h after treatment, and one day after the freezing stress treatment for physiological measurements and expression analysis. For other stress treatments, the seedlings at the four-leaf stage were sprayed on leaves with 10 mM ABA solution for ABA treatment, watered to substrate with 15% PEG6000 solution for drought stress, continuous 4 °C for cold stress, watered to substrate with 150 mM L^−1^ for salinity stress, and continuous 42 °C for heat stress. The leaves samples were harvested after 0 (CK), 1, 6, 12, and 24 h and immediately stored at −80 °C until further analysis.

### 4.2. Cloning and Bioinformatic Analysis of the BnaMYBL17 Gene

To obtain a more accurate picture of *BnaMYBL17*, a pair of degenerate primers were designed based on the conserved residues of the R2R3-MYB domain (Table S1), and the complete coding sequence was cloned using cDNA isolated from the leaves of ZS6. The reference sequence information was retrieved from *Brassica napus* Genome Browser (https://www.genoscope.cns.fr/brassicanapus/, accessed on 1 December 2022) and TAIR (https://www.arabidopsis.org/, accessed on 1 December 2022) databases. DNAMAN software (LVersion 5.2.2.; Lynnon Biosoft, Vaudreuil, QC, Canada) was used to perform the sequence alignment.

### 4.3. Expression Analysis

Using the abovementioned samples, total RNA extraction was extracted using TransZol Up Plus RNA Kit (TransGen Biotech, Beijing, China) according to the manual instructions. EasyScript^®^One-Step cDNA Synthesis SuperMix (TransGen Biotech, Beijing, China) was used to synthesize the first-strand cDNA using total RNA. The quantitative real-time PCR (qRT-PCR) was performed using an SYBR^®^Green Premix kit according to the manufacturer’s instructions on a StepOnePlusReal-Time PCR System (Applied Biosystems, Pleasanton, CA, USA). The reaction conditions were as follows: 95 °C for 10 min, followed by 40 cycles of 95 °C for 15 s and 60 °C for 30 s. The *BnaACTIN* gene was used as an endogenous reference gene. The relative expression level was determined by using the 2^−ΔΔCt^ method based on three biological repetitions. The primers used in this study are listed in Table S1.

### 4.4. Vector Construction and Plant Transformation

To construct cauliflower mosaic virus (CaMV) 35S::*BnaMYBL17*, the cDNA fragment of *BnaMYBL17* was ligated into the terminal vector pCAMBIA1300 downstream to the 35S promoter by homologous recombinase (Novozyn, Tianjin, China). Then, the resulting vector was introduced into *Agrobacterium tumefaciens* strain GV3101. To generate *BnaMYBL17*-overexpressing (OE) lines, the hypocotyl of rapeseed was transformed with 35S::*BnaMYBL17* vector using the agrobacterium-mediated transformation method to generate callus and seedlings. The *BnaMYBL17* OE lines were selected by screening of kanamycin resistance and PCR. The relative expression of *BnaMYBL17* in OE-transgenic lines was performed by qRT-PCR.

### 4.5. Observation of Subcellular Localization

To explore the subcellular localization of *BnaMYBL17*, the coding region without the stop codon was amplified using high-fidelity enzyme (Novozyn, China), and cloned into the vector pYJGFP fluorescent protein to generate 35S::BnaMYBL17::GFP construct. The pYJGFP-BnaMYBL17 plasmid was transiently expressed in the Arabidopsis protoplasts via the reported method of Zheng et al. [63]. The fluorescence of GFP was monitored using a confocal laser scanning microscope (FV1200MPE Olympus, Tokyo, Japan).

### 4.6. Transcriptional Activation and Yeast One-Hybrid Assay

The full-length CDS of *BnaMYBL17* was introduced into the pGBKT7 vector. The empty pGBKT7 plasmid served as the negative control. Then the constructs were transformed into yeast strain (AH109) following the manufacturer’s protocol. After culturing at 28 °C for three days on -Leu/-Trp medium, the survival colonies were transferred to synthetically defined (SD)/-Ade/-His/-Leu/-Trp medium to observe the transcriptional activation of *BnaMYBL17*.

For the yeast one-hybrid (Y1H) assay, the CDS sequence of *BnaMYBL17* was constructed into the pGADT7Rec vector. Four promoters of putative targets were amplified and inserted into the pHIS2.1 reporter vector. pGADT7Rec-BnaMYBL17 and the pHIS2.1 vectors with the putative MYB binding sequences were co-transformed into the yeast strain Y187 (Clontech, Mountain View, CA, USA). After being grown on SD/-Trp/-Leu selection medium for 2 days, the survival colonies were transferred to SD/-Trp/-Leu/-His selection medium containing 30 mM 3-AT to investigate the interaction strength between *BnaMYBL17* and targets’ promoters.

### 4.7. Physiological Index Determination

To study the physiological changes in rapeseed under freezing treatment, the contents of soluble sugar, proline, malondialdehyde (MDA), and relative electrolyte leakage (REL) were determined. REL was measured by a digital conductometer DDS11A (Leici Instrument Factory, Shanghai, China), according to a previous study [64]. Soluble sugar content was measured as described in the kit (cat no. G0501W; Gerusi, Wuhan, China). The proline content was measured by the sulfosalicylic acid–ninhydrin method (cat no. G0111W; Gerusi, Wuhan, China), and MDA was determined by the thiobarbituric acid (TBA) reaction as described in the kit (cat no. G0109W; Gerusi, Wuhan, China). All the physiological indexes were determined using three biological replicates and a spectrophotometer microplate reader (Epoch, BioTek, Instruments, Inc., Winooski, VT, USA).

### 4.8. RNA-seq Analysis

The RNA-seq analysis was conducted as described previously [36]. Briefly, the total RNA of three independent transgenic lines of *BnaMYBL17* (OE12, OE11, and OE9) and the wild type (WT) plants was extracted. A total of 12 high-quality RNA samples were sequenced using the Illumina Novaseq 6000 platform (Bena, Wuhan, China). Paired-end reads were generated, and clean reads were obtained after filtering. Read pairs were aligned to the rapeseed genome (*Brassica napus* Genome Browser, https://www.genoscope.cns.fr/brassicanapus/, accessed on 1 December 2022), and DEGs were evaluated by comparing three OE lines with the WT plants. HTSeq software (Version 0.9.1; European Molecular Biology Laboratory, Heidelberg, Germany) was used to calculate the expression levels of genes and transcripts, and the DEGseq method was used to detect the DEGs. The screening threshold was log2|fold change| > 1 and Q value < 0.05. Functional annotation and enrichment analysis of the DEGs were performed using the Blast2GO program.

### 4.9. Statistical Analysis

One-way analysis was conducted with SPSS version 13.0 (SPSS Inc., Chicago, IL, USA) software using Duncan’s multiple range tests at the 0.05 confidence level. The results from three independent biological replications are presented as the means ± SDs.

## 5. Conclusions

Rapeseed is one of the important oilseed crops; however, various abiotic stresses, including cold stress, significantly hinder its growth and production. MYB TFs play a vital role in understanding plant stress responses and tolerance mechanisms; however, their functional role against cold stress, mainly in rapeseed, was yet to be fully explored. Therefore, we functionally characterized an MYB *BnaMYBL17* in rapeseed under cold stress. In short, we found that *BnaMYBL17* plays a negative role in regulating freezing response in rapeseed by modulating the expression of target genes (Figure 6). *BnaMYBL17* binds to the promoter of *PLC1*, *FLZ8,* and *KOIN* to regulate the expression levels of target genes. Our results imply that *BnaMYBL17* could be a promising target for molecular breeding to improve freezing tolerance in rapeseed. The findings promote a better understanding of the molecular mechanisms underlying cold stress response in rapeseed, which is necessary for advancing the production and yield of rapeseed against adverse abiotic stress conditions.

## Data Availability

The data presented in the study are deposited in the National Center for Biotechnology Information (NCBI) repository, accession number SUB13171728, and some of the data are also available in Supplementary Materials.

## Supplementary Materials

The supporting information can be downloaded at: https://www.mdpi.com/article/10.3390/ijms24119514/s1.
