# Supplementary figures and images for: Cloning and Functional Characterization of Cold-Inducible MYB-like 17 Transcription Factor in Rapeseed (Brassica napus L.)
Source: Int J Mol Sci. 2023 May 30;24(11):9514. doi: 10.3390/ijms24119514 (PMC10253780; doi:10.3390/ijms24119514)

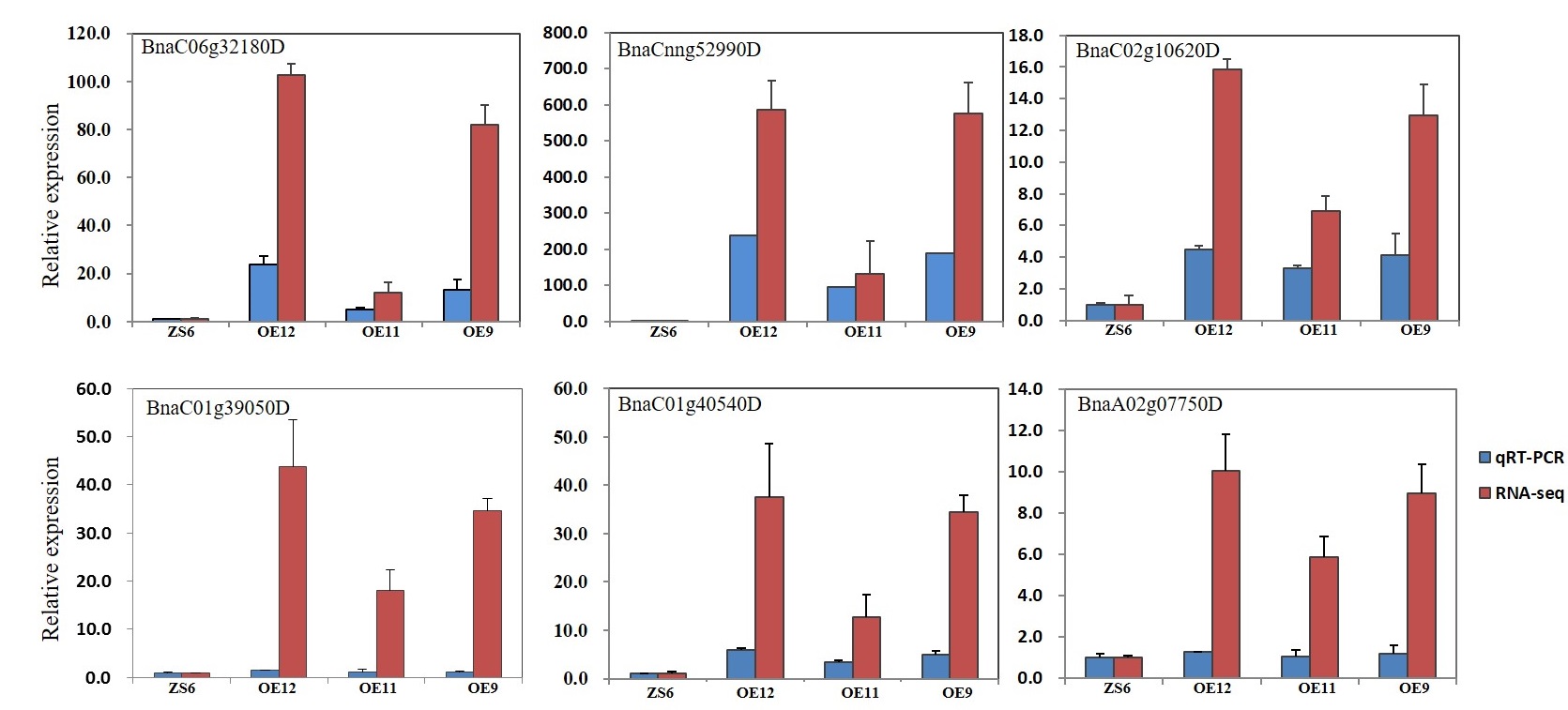

Supplement: Supplementary file 1 [file ijms-24-09514-s001.zip › Figure S1.jpg]
